# Supplementary material for: TMEM16A Maintains Acrosomal Integrity Through ERK1/2, RhoA, and Actin Cytoskeleton During Capacitation
Source: Int J Mol Sci. 2025 Apr 16;26(8):3750. doi: 10.3390/ijms26083750 (PMC12027809; doi:10.3390/ijms26083750)
Supplement: Supplementary file 1 [file ijms-26-03750-s001.zip › ijms-3529707-supplementary.pdf]

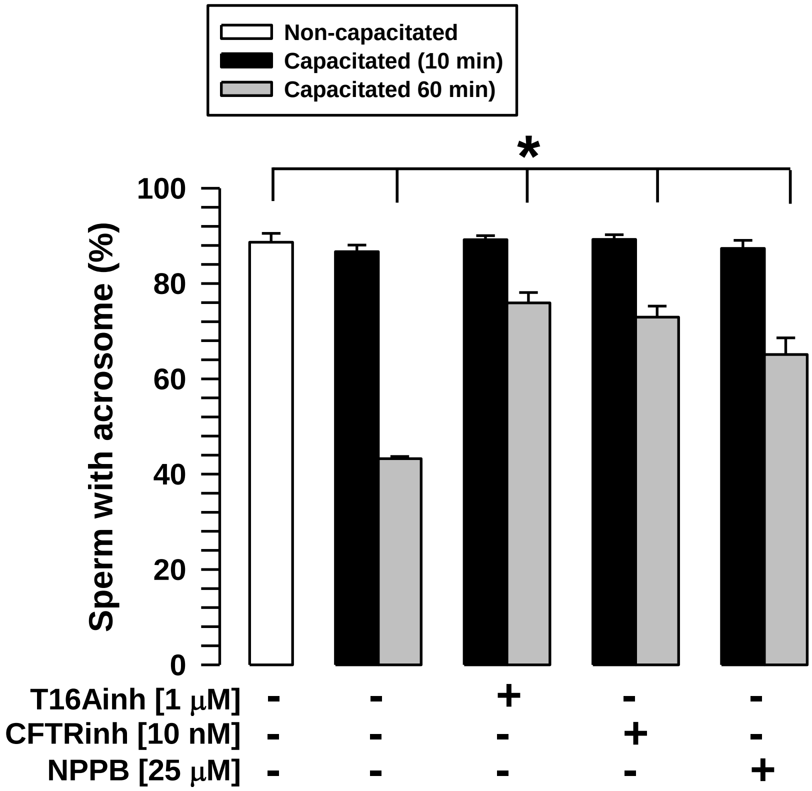

Supplementary Figure S1. Effects of Cl^-, TMEM16A, CFTR, and ClC3 channel inhibition on acrosome presence. The effects of TMEM16A, CFTR, and ClC3 channel inhibition on acrosome presence were assessed in sperm capacitated for 10 or 60 min in the absence or presence of the inhibitors T16Ainh, CFTRinh, or NPPB. Acrosome presence was determined by CTC staining. Results are expressed as mean ± SE (n = 3), *p <0.05.
